# Physcion isolated from *Senna alata*-mediated green synthesis of ZnO nanoparticles: mechanistic and comparative evaluation for photocatalytic doxycycline degradation

**DOI:** 10.1039/d6ra01923d

**Published:** 2026-07-02

**Authors:** Khieu Thi Tam, Cao Thanh Hai, Dang Van Thanh, Nguyen Khac Tung, Le Tien Ha, Tran Trung Hieu, Dinh Thi Hong Minh, Vu Thi Hue, Vuong Truong Xuan

**Affiliations:** a TNU-University of Sciences Phan Dinh Phung Ward Thai Nguyen 25000 Vietnam xuanvt@tnus.edu.vn; b Thai Nguyen University of Pharmacy and Medicine Thai Nguyen 25000 Vietnam; c VNU Key Laboratory of Green Environment, Technology and Waste Utilization (GreenLab), University of Science, Vietnam National University Hanoi Vietnam; d Department of Chemistry, Vinh University Truong Vinh Nghean 43000 Vietnam; e Faculty of Basic Sciences, Vietnam University of Traditional Medicine (VUTM) No. 2, Tran Phu Street, Dai Mo Ward Hanoi Vietnam; f Thai Binh University of Medicine and Pharmacy Tran Lam Ward Hung Yen Vietnam

## Abstract

Green synthesis of ZnO nanoparticles (ZnO NPs) using plant-derived compounds has emerged as an attractive alternative to conventional chemical methods. Despite extensive interest in phytochemical-assisted synthesis, the molecular processes governing nanoparticle nucleation and growth remain incompletely understood. In the present work, ZnO NPs prepared using crude *Senna alata* extract (SAZ) and its major anthraquinone constituent, physcion (PZ), were compared with chemically synthesized ZnO (Z) to examine how a structurally defined phytochemical influences ZnO formation. FTIR and HPLC analyses, together with complementary structural characterization, indicated that physcion participates in coordination interactions with Zn^2+^ species and remains involved throughout nanoparticle development. Its presence was associated with more controlled crystallite growth, surface stabilization, and the formation of quasi-spherical ZnO nanoparticles with a smaller crystallite size (14.74 nm) and a higher specific surface area (40 m^2^ g^−1^) than those obtained from crude extract- or chemical-mediated synthesis. Photoluminescence measurements further suggested reduced charge-carrier recombination in PZ, consistent with differences in surface and defect-related environments. Among the investigated materials, PZ exhibited the highest photocatalytic performance, achieving 81.02% doxycycline degradation under UVA irradiation together with an enhanced apparent reaction rate constant. The comparison between physcion and the crude plant extract illustrates how the use of a single, structurally defined phytochemical can influence ZnO nucleation, crystal growth, and surface evolution more effectively than chemically heterogeneous botanical mixtures. These findings provide molecular-level insight into anthraquinone-mediated ZnO formation and highlight the value of selected phytochemicals as tools for the rational design of green photocatalysts for antibiotic remediation.

## Abstract 2

Green synthesis of ZnO nanoparticles (ZnO NPs) using plant-derived compounds has emerged as an attractive alternative to conventional chemical methods.

## Graphical abstract

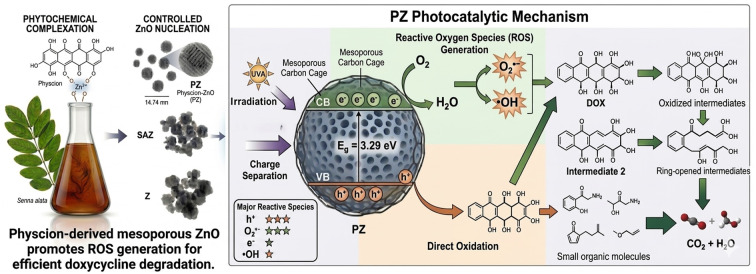

## 1. Introduction

Photocatalysis based on semiconductor materials remains one of the practical routes for degrading persistent organic contaminants in water systems.^1,2^ Among the available candidates, zinc oxide (ZnO) continues to receive attention owing to its wide band gap, photochemical stability, low cost, and relatively low toxicity.^3,4^ Traditional preparation routes, though reliable, frequently require strong alkaline media, elevated temperatures, or additional stabilizing chemicals.^5,6^ Such conditions are not fully aligned with current sustainability priorities.

Plant-mediated synthesis has emerged as an alternative pathway in which naturally occurring organic molecules participate in metal ion coordination, hydrolysis regulation, and nanoparticle stabilization.^7,8^ Within these biomolecules, anthraquinones are structurally distinctive because of their conjugated quinone core and adjacent hydroxyl groups.^9,10^ These functional moieties readily coordinate with Zn^2+^ species and may influence hydrolysis, condensation, and crystal growth behavior during ZnO formation.^11^

Most reports on green ZnO synthesis rely on crude plant extracts. Extracts contain dozens of metabolites with overlapping redox and chelation properties. Under such conditions, assigning a clear mechanistic role to any single compound becomes difficult. As a consequence, correlations between phytochemical composition and nanoparticle characteristics are often descriptive rather than mechanistically grounded.^12,13^ Questions remain regarding how hydroxyl and carbonyl groups interact with Zn^2+^ during early-stage complexation, how these interactions modify nucleation kinetics, and whether selective crystal facet development can be directed through defined molecular coordination.^14^ Limited molecular-level evidence continues to constrain predictive control over particle size, morphology, and defect formation in green ZnO systems.

Isolating a single, structurally defined phytochemical provides a more controlled framework for addressing these uncertainties. Anthraquinones, owing to their redox-active quinone backbone and metal-binding hydroxyl groups,^9,10^ offer a suitable platform for such investigation. However, systematic evaluation of an isolated anthraquinone compound in ZnO nucleation and growth has seldom been reported. Direct molecular-level evidence linking phytochemical transformation to ZnO formation therefore remains limited. Consequently, a detailed understanding of the green synthesis mechanism of ZnO is essential for the rational design of nanoparticles with tailored structural properties. Particular attention should be given to the sequence of processes involved, including Zn^2+^-biomolecule complex formation, nucleation, crystal growth, and nanoparticle aggregation.^15^ A comprehensive understanding of the relationship between the chemical structure of natural anthraquinones, synthesis conditions, and the resulting morphological and surface characteristics of ZnO not only improves the reproducibility of green synthesis methods but also enables rational tuning of ZnO performance for applications such as photocatalysis and biomedical uses.^3^

The medicinal plant *Senna alata* contains abundant anthraquinone derivatives.^16,17^ Physcion, one of its principal constituents, carries both phenolic hydroxyl and quinone functionalities capable of participating in electron transfer and metal coordination.^18^ These structural features suggest a dual role during nanoparticle formation: transient complexation with Zn^2+^ and surface stabilization of the growing oxide phase. Detailed clarification of this role is still lacking, particularly in relation to structural evolution and photocatalytic behavior.

Parallel to these materials considerations, antibiotic contamination continues to challenge aquatic environments. Doxycycline (DOX), a tetracycline-class antibiotic widely used in clinical and agricultural settings,^19,20^ persists in water bodies due to incomplete biodegradation. Its accumulation contributes to resistance development and ecological disturbance.^21,22^ Photocatalytic degradation offers one remediation pathway, yet catalytic efficiency depends strongly on nanoparticle structure and surface chemistry.

In the present work, physcion isolated from *Senna alata* is employed as a defined molecular mediator in ZnO synthesis. Nanoparticles prepared using crude extract (SAZ), isolated physcion (PZ), and conventional co-precipitation (Z) are examined in parallel. Spectroscopic and chromatographic analyses are used to track chemical transformations of physcion during ZnO formation, enabling assessment of Zn^2+^ coordination, redox modification, and surface stabilization effects. Structural characteristics are subsequently correlated with photocatalytic degradation of DOX under UV irradiation. Through this comparative framework, the influence of a single anthraquinone compound on ZnO nucleation, morphology, and catalytic behavior is examined under controlled conditions, providing a clearer connection between phytochemical chemistry and functional nanomaterial design.

## 2. Experiments

### 2.1. Materials

All chemicals used in the experiments were of analytical grade. Zinc nitrate hexahydrate (Zn(NO_3_)_2_·6H_2_O, ≥98%) and sodium hydroxide (NaOH, ≥99.7%) were employed in the synthesis of ZnO NPs. Doxycycline was selected as a representative pollutant for evaluating photocatalytic performance. Silver nitrate (AgNO_3_, ≥99%), ethylenediaminetetraacetic acid (EDTA, ≥99%), ascorbic acid (≥98%), and *tert*-butanol (≥99%) were trapping agents.

### 2.2. Chemical constituents of *Senna alata*

To identify the main compounds in *Senna alata* responsible for the formation of ZnO NPs, the isolation and structure determination of compounds were performed. 3 kg of *Senna alata* dried powder was sonicated with absolute ethanol at 30 °C. 200 mL of the ethanol extract was stored at 4 °C for green synthesis of *Senna alata*-ZnO NPs, while the remaining extract was evaporated to remove the solvent and obtain the crude ethanolic extract. The crude ethanolic extract was suspended with water, then partitioned with *n*-hexane, ethylacetate (EtOAc) and *n*-butanol to gain the corresponding *n*-hexane, ethylacetate and *n*-butanol extracts, respectively. The obtained extracts were examined by thin-layer chromatography (TLC) to evaluate the chemical compositions. Thin-layer chromatography was carried out on precoated silica gel 60 F_254_ plates. The chromatography was visualized under a UV lamp at 254 and 365 nm and spots were further detected by spraying with reagents. After examining the TLC, the ethylacetate extract was subjected to column chromatography over silica gel (60 G, 0.043–0.063 nm particle size), eluting with a gradient *n*-hexane : EtOAc (100 : 0–0 : 100, v/v) to furnish 8 fractions (E1–E8). Fraction E4 was rescrystallized with dichloromethane to yield a pure compound (78.6 mg). The structure of the compound was determined by FTIR, ^1^H-NMR and compared with reported data. The FT-IR spectra (Spectrum Two, Perkin Elmer, USA) were measured in a wavenumber range of 4000–450 cm^−1^ to determine the presence of functional groups. NMR spectra were measured by a Bruker Advance I (600 MHz), CH_3_OD was used as solvent for NMR analysis.

### 2.3. Synthesis of ZnO NPs (SAZ, PZ and Z)

Zinc nanoparticles were synthesized *via* three different routes, including green synthesis using *Senna alata* extract (SAZ), physcion-mediated synthesis (PZ) and conventional co-precipitation (Z). In all synthesis routes, 500 mL of 0.1 M Zn(NO_3_)_2_ solution was used as the zinc precursor. For the SAZ sample, 50 mL of *Senna alata* ethanol extract was added to the Zn(NO_3_)_2_ solution under continuous stirring. The phytochemical constituents present in the extract were expected to participate in Zn^2+^ coordination and nanoparticle stabilization during synthesis.^23^

**Fig. 1:**
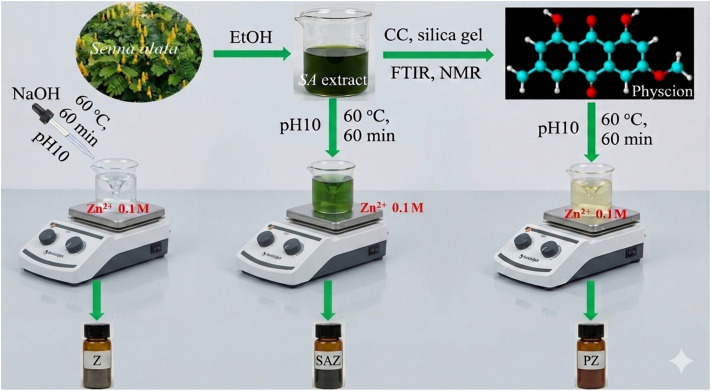
Schematic illustration of the synthesis procedures for Z, SAZ and PZ.

For the PZ sample, physcion was employed as a single, well-defined phytochemical to investigate its role in ZnO formation. Physcion was dissolved in an ethyl acetate–methanol mixture (50 : 50, v/v) to prepare a 0.01 M solution. Subsequently, 10 mL of the physcion solution was added dropwise to the Zn(NO_3_)_2_ solution under continuous stirring. For comparison, ZnO NPs (Z) were prepared by a classical co-precipitation method using NaOH. All reactions were conducted at pH 10 and stirred for 60 minutes at 60 °C. The reaction mixture was subsequently filtered and washed with distilled water and ethanol until neutral, then dried and ground for further experiments. After filtration, the reaction solution from the PZ synthesis was evaporated to dryness to obtain a solid residue. The solid product was subsequently characterized by FTIR and HPLC analyses to elucidate the reaction mechanism involved in the synthesis of ZnO NPs. The schematic diagram for the synthesis of SAZ, PZ and Z was displayed in Fig. 1.

### 2.4. Characterization of SAZ, PZ and Z

The characteristics of SAZ, PZ and Z were investigated by FTIR spectra, X-ray diffraction (XRD), scanning electron microscopy (SEM), energy-dispersive X-ray spectra (EDS), transmission electron microscopy (TEM), Brunauer–Emmett–Teller (BET), ultraviolet visible diffuse reflectance spectroscopy (UV-vis DRS), photoluminescence (PL) and PL decay times. FTIR spectra were recorded using a Spectrum two spectrometer (Perkin Elmer, USA) in the wavenumber range of 4000–500 cm^−1^ to identify functional groups. The crystal structure of samples was measured by XRD (D2-Phaser, Brucker, Japan) at a 2*θ* range of 20° to 80°. The crystalline size (*D*) was calculated by the Debye Scherrer formula using eqn (1).1
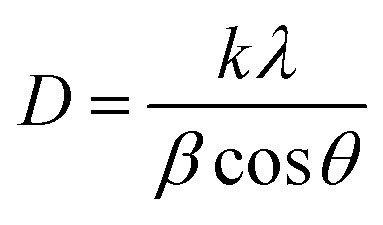
where *D* is the crystalline size (nm), *k* is the Scherrer constant (*k* = 0.9), *λ* is the X-ray wavelength (*λ* = 0.15406), *β* is the full width at half maximum (radians), and *θ* is the angle of diffraction (radians).

The surface morphology and elemental compositions of all samples were examined by SEM and EDS spectroscopy using a JSM-IT100 instrument (Jeol, Tokyo, Japan). The additional structural details were analyzed by TEM images using a JEOL 2100F microscope (Tokyo, Japan). The specific surface area was determined using N_2_ adsorption/desorption curves (BET, Tristar-300, Norcross, USA). The optical properties of the samples were investigated using UV-vis DRS (S-4100, Scinco, Seoul, Korea) in the wavelength ranging from 300 nm to 800 nm and PL spectroscopy, which was recorded on a NanoLog spectrophotometer (Horiba) with a 450 W xenon lamp, while PL decay times were obtained from an Agilent Cary Eclipse fluorescence spectrophotometer. The chemical bonding configuration of PZ sample was analyzed on X-ray photoelectron spectroscopy (XPS, Nexsa G2).

### 2.5. Photocatalytic degradation of doxycycline

Photocatalytic degradation experiments were conducted under UVA irradiation using a Philips TL 8W BLB lamp (8W, 356 nm) with an irradiation intensity of 2.02 mW cm^−2^ at the sample surface. The lamp was positioned 10 cm above the sample surface, and all experiments were performed in a dark chamber maintained at 25 °C to ensure uniform light exposure and minimize interference from external light sources. A total of 350 mL of DOX solution (10 mg L^−1^) was transferred to a tube containing 0.35 g of photocatalyst (SAZ, PZ and Z). The initial pH of the reaction solution was adjusted to 5 using NaOH or HCl solutions.^23^ Prior to irradiation, the suspension was stirred in the dark for 30 min to establish adsorption–desorption equilibrium. Subsequently, the mixture was exposed to UVA irradiation under continuous magnetic stirring for 150 min. At predetermined time intervals (every 30 min), 10 mL aliquots were withdrawn and centrifuged at 7000 rpm for 10 min to remove catalyst. The residual concentration of DOX was determined by measuring the absorbance at 370 nm using a Hazch UV-vis spectrophotometer. All experiments were carried out in triplicate to ensure reproducibility. The degradation efficiency (%) of DOX was calculated according to the eqn (2).2
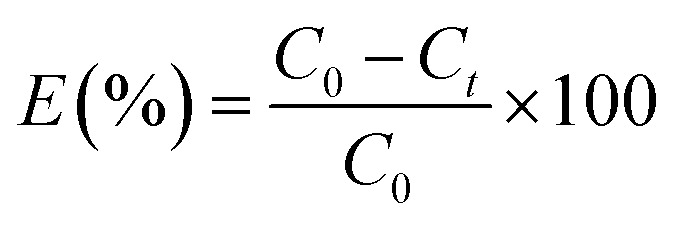
where *C*_0_ and *C*_*t*_ were the concentrations of DOX at initial and *t* times, respectively.

First-order kinetics was studied to identify the degradation rate of DOX after obtaining the optimal conditions using equilibrium (3) analysis.3
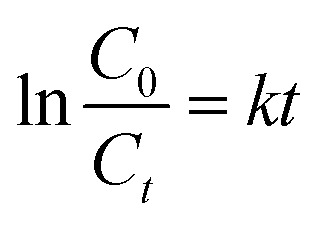
where *k* was the first-order rate constant, and *t* was the reaction time.

To evaluate the scavenging ability of reactive oxygen species (ROS), 4.5 mL of AgNO_3_, EDTA, ascorbic acid and *tert*-butanol was utilized as a scavenger for e^−^, h^+^, O_2_˙^−^ and ˙OH, respectively. Each scavenger was added to the 350 mL of the reaction solution at a concentration of 1 mM. After completion of the photocatalytic experiment, the catalysts were collected and thoroughly washed multiple times with double-distilled water before being reused in subsequent cycles under identical reaction conditions.

To determine the products formed after the reaction, the solution obtained after photocatalytic degradation was then evaporated in a vacuum to yield a solid. The solid was then used to analyze DOX degradation products using the FTIR spectrum.

## 3. Results and discussions

### 3.1. Main chemical constituents of *Senna alata* and the mechanism for green synthesis of ZnO NPs

Plant extracts contain chemically diverse constituents, and different phytochemicals may play distinct roles during nanomaterial synthesis. Therefore, identification of the major phytochemical components represents an important step toward understanding the mechanisms governing nanoparticle formation, particularly because such compounds may participate in metal-ion coordination, regulation of hydrolysis and nucleation processes, crystal growth control, and surface stabilization. Improved knowledge of these interactions may also provide insight into factors influencing nanoparticle size, morphology, and functional performance.

**Fig. 2:**
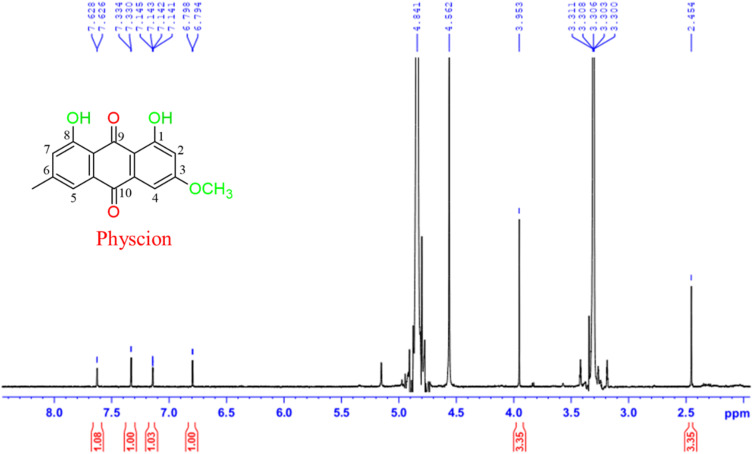
^1^H-NMR spectra of physcion isolated from *Senna alata*.

Following TLC screening and chromatographic separation, the major compound in the extract was isolated and identified as physcion. The structure of physcion was determined using FTIR and ^1^H NMR spectroscopy and further confirmed by comparison with previously reported spectral data. As shown in Fig. 2 and S1 SI, the ^1^H NMR spectrum exhibited a singlet at *δ* 2.45 ppm (3H), corresponding to the aromatic methyl group at the 6-position (6-CH_3_), consistent with the characteristic structure of physcion.

Another singlet observed at *δ* 3.95 ppm (3H) was attributed to the methoxy group (3-OCH_3_). Four aromatic proton signals characteristic of the anthraquinone skeleton of physcion were observed at *δ* 6.79 (H-7) and 7.14 ppm (H-2), 7.33 (H-5) and 7.63 ppm (H-4). The phenolic hydroxyl protons at C-1 and C-8 were not observed, likely due to rapid exchange with CD_3_OD.

**Fig. 3:**
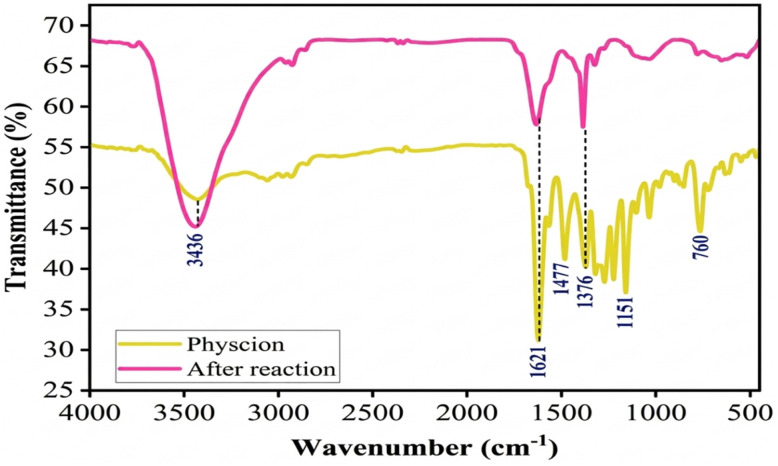
The FTIR spectra of physcion and the mixture after reaction.

The FTIR spectrum of physcion, as seen in Fig. 3, exhibited a broad absorption band at 3436 cm^−1^, which was attributed to the stretching vibration of phenolic hydroxyl (–OH) groups, consistent with the anthraquinone structure bearing free hydroxyl functionalities. The strong peak observed in the range of 1620–1660 cm^−1^ was assigned to the conjugated C=O stretching vibrations of the anthraquinone system, with partial overlap from aromatic C=C stretching vibrations, which is a typical FTIR feature of anthraquinone derivatives. Furthermore, absorption peaks at 1477 and 1376 cm^−1^ were assigned to aromatic C=C stretching and C–H bending vibrations, respectively, while the absorption peak at 1151 cm^−1^ was associated with C–O stretching vibration. The absorption peak near 760 cm^−1^ was attributed to out of plane bending vibration of the aromatic ring. Thus, the structure of the isolated compound from *Senna alata* was 1,8-dihydroxy-3-methoxy-6-methylanthraquinone or physcion, which was determined by spectroscopic analysis and compared with published data.^24–26^ Physcion is a natural, orange yellow anthraquinone derivative available in many herbs like *Senna alata*, *Vitis vinifera* and *Rhizoma graminis*.^18,27^ The reducing capability of physcion arises from the conjugation of phenolic hydroxyl groups with a redox-active anthraquinone backbone,^26^ enabling effective coordination-mediated nucleation control.^27,28^ This structural arrangement facilitates coordination interactions between physcion and Zn^2+^ ions through hydroxyl and carbonyl functionalities, while the extended π-electron system contributes to the stabilization of coordination complexes and surface-bound species.^29^ These properties render physcion an effective coordination-active stabilizing and structure-directing agent in green ZnO nanoparticle synthesis. As a result, physcion played a crucial role in the green synthesis of ZnO NPs using *Senna alata* extracts. Thus, ZnO NPs were synthesized using both crude *Senna alata* extract and its isolated major constituent, physcion.

FTIR and HPLC analyses were used to evaluate the role of physcion during the green synthesis of ZnO NPs and to confirm the product generated from physcion after the reaction. The FTIR spectroscopy of the reaction mixture after removal of ZnO NPs (Fig. 3) revealed marked spectral changes, indicating a direct participation of physcion in the ZnO formation process. The intensity of broad –OH stretching vibrations at 3436 cm^−1^ was significantly decreased and slightly shifted, suggesting deprotonation and coordination of phenolic hydroxyl groups with Zn^2+^ during nanoparticle formation.

Notably, the absorption peak at 1621 cm^−1^, assigned to the conjugated C=O stretching vibrations of the anthraquinone moiety, became markedly weaker, implying the involvement of quinone carbonyl groups in coordination interactions and possible redox-related transformation during the reduction process. Furthermore, absorption peaks at 1477 and 760 cm^−1^, which were attributed to C=C stretching vibrations and =CH bending vibrations of aromatic rings, disappeared, indicating structural modification of aromatic system during ZnO formation. In addition, significant variations in the intensity of absorption peaks at 1376 and 1151 cm^−1^ indicated chemical changes in physcion, which may be associated with coordination interactions, possible formation of C–O–Zn linkages and partial oxidative transformation of the anthraquinone skeleton.

**Fig. 4:**
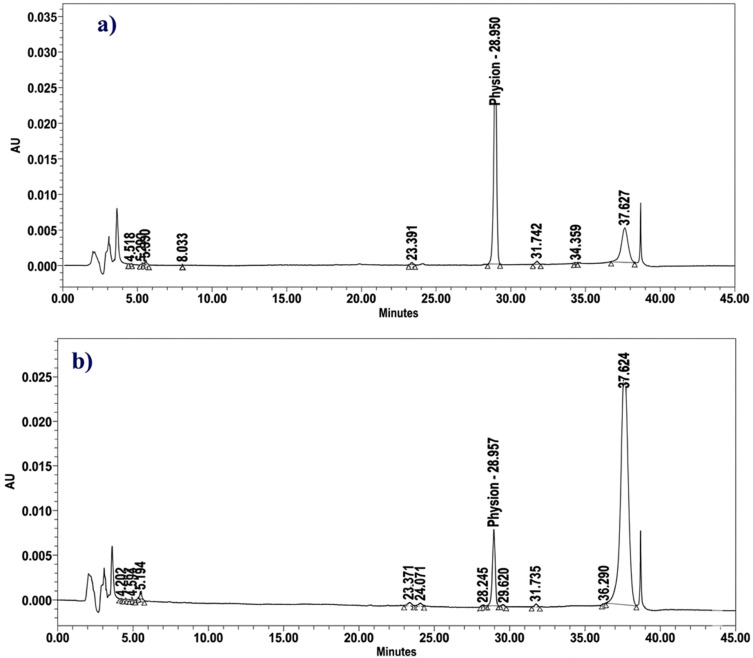
HPLC chromatography of (a) physcion and (b) mixture after reaction.

The HPLC chromatogram of pure physcion (Fig. 4a) exhibited a dominant peak at a retention time of 28.950 min together with a minor peak at 37.627 min. Following the synthesis process and subsequent removal of the PZ nanoparticles, the chromatographic profile of the remaining reaction mixture changed markedly (Fig. 4b). The intensity of the principal physcion peak at 28.957 min decreased substantially, while the peak at 37.624 min became significantly more prominent.

These chromatographic changes indicate substantial consumption and transformation of physcion during the ZnO synthesis process and suggest the formation of reaction-derived species with chromatographic characteristics distinct from those of the parent compound.^30^ The pronounced reduction of the major physcion peak further indicates that physcion participates in the reaction system rather than remaining as a spectator component. When considered together with the FTIR results, which revealed alterations in hydroxyl- and carbonyl-related vibrational bands, the HPLC data suggest that physcion undergoes chemical interactions during ZnO nanoparticle formation, consistent with the known chemical reactivity and transformation pathways of quinone-based compounds reported in previous studies.^28^ Although the exact structures of the transformed species cannot be established solely from the present chromatographic data, the combined FTIR and HPLC evidence is consistent with the participation of physcion in the nanoparticle formation process. Accordingly, physcion likely contributes to the chemical environment associated with ZnO nanoparticle nucleation and growth rather than merely serving as an inert additive.

### 3.2. Characterizations of SAZ, PZ and Z

**Fig. 5:**
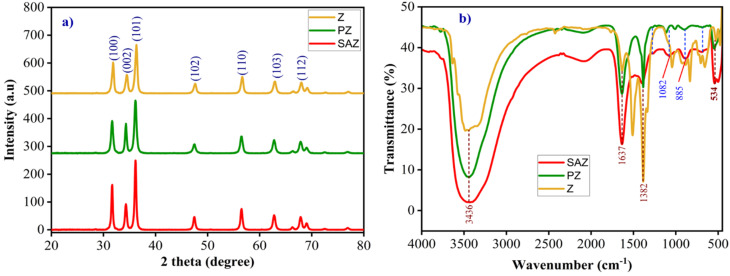
(a) XRD of SAZ, PZ and Z; (b) FTIR spectroscopy of SAZ, PZ and Z.

The crystalline structure and phase purity of synthesized ZnO NPs *via* different routes, including SAZ, PZ and Z, were investigated by XRD. As shown in Fig. 5a, all samples exhibited diffraction peaks at 2*θ* values of approximately 31.61°, 34.29°, 36.18°, 47.44°, 56.54°, 62.86°, 66.37°, 67.94°, 69.08°, 72.55° and 76.93°, which can be indexed to the (100), (002), (101), (102), (110), (103), (200), (112), (201), (004) and (202) crystallographic planes of the hexagonal wurtzite ZnO structure, in good agreement with the standard PDF card (JCPDS No. 80-0075), respectively. No additional peaks corresponding to impurity phases such as Zn(OH)_2_, metallic Zn, or other zinc oxides were detected, confirming the successful formation of single-phase ZnO in all samples.

The average crystallite sizes of SAZ, PZ and Z calculated using eqn (1) were 18.16 nm, 14.69 nm and 16.51 nm, respectively. Furthermore, a comparative analysis of peak intensity and broadening revealed that the synthesis route significantly influenced the crystallinity of ZnO NPs. Compared with the Z sample, the SAZ and PZ samples exhibited relatively broadened diffraction peaks with slightly reduced intensities, suggesting that the presence of organic species during synthesis affected the crystallization process of ZnO. In contrast, the Z sample displayed sharp and intense diffraction peaks, indicating a higher degree of crystallinity and well-developed crystal structure.

These differences can be attributed to the presence of organic compounds during synthesis, which may act as capping and stabilizing agents, partially limiting particle growth, potentially introducing lattice distortions and structural defects.^31,32^ Notably, the PZ sample showed more pronounced peak broadening than SAZ, consistent with its smaller crystallite size. This result suggested that physcion may influence the nucleation and growth of ZnO crystals through coordination interactions with Zn^2+^ ions. The coordination interaction likely reduced the crystal growth rate while promoting homogeneous nucleation, leading to the formation of smaller crystallites. The pronounced peak broadening in the PZ sample may also be attributed to increased lattice microstrain, in addition to the reduced crystallite size. These effects likely arise from coordination-induced lattice distortion during physcion-mediated growth, leading to structural disorder such as oxygen vancacies and surface defects. In comparison, the SAZ sample exhibited a relatively larger crystallite size, which may be attributed to the less controlled coordination interactions between Zn^2+^ ions and the heterogeneous phytochemicals present in the crude extract.

Thus, the use of plant extract and physcion influenced the crystallization behavior of ZnO by modifying crystallite size and crystallinity. These structural variations were expected to influence the surface properties and defect density of ZnO NPs, which are critical factors governing their optical and photocatalytic performance.

The functional groups involved in the synthesis and stabilization of ZnO NPs were determined through FT-IR spectroscopy analysis of *Senna alata* extract, physcion, SAZ, PZ and Z, as shown in Fig. 5b and S2 (SI). The FTIR spectrum of SAZ showed an absorption peak at 534 cm^−1^, which was assigned to the Zn–O stretching vibration, confirming the successful formation of ZnO NPs.^33,34^ Importantly, characteristic absorption peaks of *Senna alata* extracts were also clearly observed in the FTIR spectrum of SAZ, indicating the incorporation of phytochemical constituents on the ZnO surface. Absorption peaks at 3436 cm^−1^ for O–H stretching vibrations, along with the peak at 1637 cm^−1^ for stretching vibration of aromatic C=C bond, conjugated C=O bonds were retained in the SAZ spectrum. In addition, absorption peaks at 1382 cm^−1^, 1082, and 885 cm^−1^, associated with C–O stretching and =CH bending vibrations, were also detected in the SAZ spectra.^35^ However, the intensity of these peaks decreased. The results indicate that the phytochemical constituents of *Senna alata* extract actively participated in the formation of ZnO nanoparticles through coordination interactions with Zn^2+^ ions and regulation of nucleation and crystal growth processes. In addition, some phytochemical species remained associated with the ZnO surface, contributing to surface stabilization and passivation. The FTIR spectroscopy of PZ exhibited characteristic absorption peaks of physcion including peaks at 3436, 1621 and 1477 cm^−1^ with lower intensities, along with an absorption peak at 534 cm^−1^, which was associated with the stretching vibration of Zn–O. Overall, the FTIR results suggest that physcion functioned primarily as a coordination-active, chelating, and stabilizing agent during the green synthesis of ZnO. The phenolic hydroxyl and quinone-type carbonyl groups of physcion likely facilitated coordination with Zn^2+^ species, thereby influencing hydrolysis, nucleation, and crystal growth during nanoparticle formation. In addition, these functional groups may interact with surface Zn sites, potentially contributing to surface passivation and enhanced particle stabilization of the resulting ZnO nanoparticles. Furthermore, coordination interactions between Zn^2+^ ions and the hydroxyl and quinone-type carbonyl groups of physcion likely influenced nucleation kinetics and restricted subsequent crystallite growth during nanoparticle formation. Compared with SAZ, the PZ sample exhibited a reduction in crystallite size from 18.16 to 14.69 nm, together with a decrease in average particle size from 32.28 to 14.74 nm based on TEM analysis. These reductions suggest that these oxygen-containing functional groups provided localized coordination sites for Zn^2+^ species, thereby moderating hydrolysis and limiting particle growth and aggregation during crystal formation. This interpretation is supported by the combined FTIR evidence for hydroxyl and carbonyl group involvement, as well as the broadened XRD diffraction peaks and smaller TEM-derived particle sizes observed for PZ relative to SAZ. The broader XRD diffraction peaks observed for PZ may reflect not only the reduction in crystallite size but also the presence of lattice microstrain and increased structural disorder within the ZnO nanocrystals. These structural features are consistent with the coordination-mediated growth mechanism discussed above and may reflect local variations in lattice environments generated during physcion-assisted crystallization. This interpretation is also consistent with the defect-related characteristics identified in the PL and XPS analyses. Direct quantification of lattice microstrain, however, was beyond the scope of the present study.

**Fig. 6:**
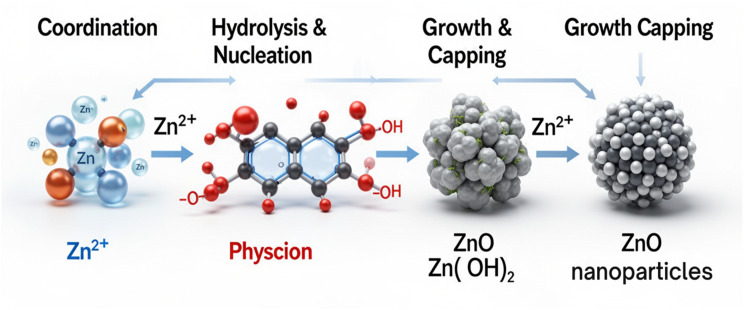
Proposed mechanism of the reaction of PZ synthesis using physcion.

Whereas the FTIR spectrum of Z displayed characteristic peaks of ZnO NPs, such as peaks at 3411, 1635, 1478 and especially, the absorption peaks at 509 cm^−1^ were assigned to the Zn–O stretching vibration. Based on the combined FTIR and HPLC evidence, together with the structural characteristics observed by XRD and TEM analyses, a plausible mechanism for physcion-mediated ZnO formation is proposed and illustrated in Fig. 6.

#### 3.2.1 Proposed mechanism of ZnO formation

To further elucidate the role of physcion during nanoparticle formation, a plausible reaction pathway for the formation of ZnO nanoparticles in the physcion-mediated system is proposed based on coordination chemistry principles and previous studies on phytochemical-assisted ZnO synthesis. The proposed formation pathway is schematically illustrated in Fig. 6.

The formation of ZnO nanoparticles in the physcion-mediated system is more reasonably interpreted through a coordination-hydrolysis-nucleation mechanism rather than a direct reduction pathway. Under alkaline conditions, Zn^2+^ ions undergo hydrolysis to form zinc hydroxide intermediates, which subsequently dehydrate to generate ZnO nuclei according to the classical pathway:Zn^2+^ + 2OH^−^ → Zn(OH)_2_ → ZnO + H_2_O

In this process, physcion primarily functions as a redox-active coordination and capping agent rather than a conventional reducing agent. The molecule contains phenolic hydroxyl and carbonyl groups capable of coordinating Zn^2+^ ions through oxygen donor atoms. Such coordination interactions can locally concentrate Zn^2+^ species and influence the hydrolysis and nucleation processes during ZnO formation. Similar interactions between polyphenolic compounds and Zn^2+^ ions have been reported in phytochemical-assisted ZnO synthesis, where organic ligands regulate nucleation and crystal growth. In addition, physcion molecules may adsorb onto the surface of the growing ZnO nanocrystals through hydrogen bonding or coordination with surface Zn sites, acting as a capping agent that suppresses particle agglomeration. The observed decrease in the physcion peak in HPLC after the reaction further suggests that the molecule participates in the synthesis process, possibly through coordination or partial transformation under alkaline conditions.

The combined FTIR, HPLC, and XPS results are consistent with the participation of physcion-derived hydroxyl and carbonyl functionalities during ZnO nanoparticle formation. FTIR revealed changes in hydroxyl- and carbonyl-related vibrational bands, while HPLC demonstrated substantial consumption and transformation of physcion during synthesis. XPS confirmed the formation of ZnO nanoparticles and revealed defect-related oxygen environments that may arise from the interaction of organic species with the growing oxide surface. Collectively, these observations support the proposed involvement of physcion in coordination-mediated ZnO formation. However, the current data do not permit unequivocal identification of specific Zn–O–C bonding configurations, and therefore the proposed Zn-physcion coordination pathway should be regarded as a plausible mechanistic interpretation rather than definitive structural proof.

The distinctive structural characteristics of PZ appear to originate from the multifaceted involvement of physcion throughout the ZnO formation process. Coordination between Zn^2+^ species and the phenolic hydroxyl and quinone-type carbonyl groups of physcion likely modified the local chemical environment during hydrolysis and early-stage nucleation, creating conditions favorable for more controlled crystal development, consistent with coordination-assisted growth mechanisms reported for phytochemical-mediated ZnO synthesis.^36,37^ At the same time, adsorption of physcion-derived species onto the surface of growing ZnO nuclei may have reduced interparticle coalescence during crystallization, limiting excessive growth and aggregation, a behavior commonly associated with the capping and stabilizing effects of oxygen-containing natural compounds during nanoparticle formation.^36–39^

This interpretation is supported by the collective structural evidence. Relative to SAZ, PZ exhibited a smaller crystallite size, reduced particle dimensions, a more uniform near-spherical morphology, and a higher specific surface area. Comparable trends have been reported for ZnO nanoparticles synthesized using structurally defined phytochemicals such as puerarin and rutin, where molecular-level regulation of nucleation and crystal growth resulted in improved control over particle morphology, crystallite size, and surface characteristics.^38,39^

The influence of physcion therefore extends beyond simple coordination with Zn^2+^ precursors. Its participation throughout nucleation, crystal growth, and particle stabilization provides a plausible explanation for the distinct structural evolution observed in PZ. The resulting architecture offers a larger accessible interfacial area and shorter transport distances for photogenerated charge carriers, structural features frequently associated with enhanced photocatalytic performance in green-synthesized ZnO nanomaterials.^40^

**Fig. 7:**
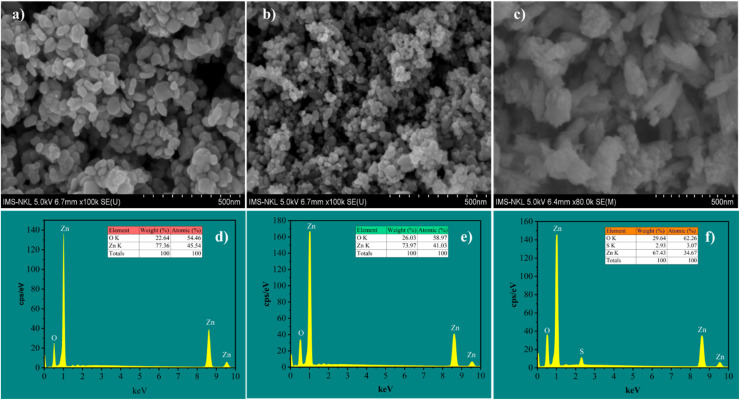
SEM images of SAZ (a), PZ (b) and Z (c) EDS of SAZ (d), PZ (e) and Z (f).

The SEM images revealed noticeable differences in the morphology of SAZ, PZ and Z samples (Fig. 7a–c). The SAZ sample consisted of ZnO NPs with quasi-spherical to short ellipsoidal morphologies, indicating a moderate degree of size uniformity. These agglomerates exhibited a relatively porous structure with visible interparticle voids. The observed aggregation was mainly attributed to the high surface energy of ZnO NPs and the heterogeneous nature of phytochemicals present in *Senna alata* extract. While these biomolecules acted as coordination-active, stabilizing, and capping agents, their mixed composition may lead to less effective surface passivation, resulting in partial particle coalescence during growth. In contrast, the PZ sample showed smaller and more uniformly distributed nanoparticles, predominantly near-spherical in shape. The nanoparticles formed finer and more homogeneous agglomerates, giving rise to a well-developed hierarchical porous structure with interconnected voids. The smoother particle surfaces and improved size uniformity suggested that physcion played a more effective role in controlling nucleation and crystal growth and limited uncontrolled aggregation.

These features were consistent with the reduced crystalline size observed in the XRD analysis. Whereas the Z sample indicated aggregated rod-like particles with a non-uniform size distribution. The boundaries between individual particles were not clearly distinguishable, indicating strong interparticle interactions. The elemental compositions of SAZ, PZ and Z were determined using EDS measurements as shown in Fig. 7(d–f). The spectrum of SAZ, PZ indicated only the presence of zinc (Zn) and oxygen (O) without any other compositions, while Z indicated the presence of sulfur (S). The quantitative analysis of all elemental constituents of SAZ, PZ and Z was summarized in the table inserted in Fig. 7d–f. The EDS spectra of SAZ and PZ contained only zinc (Zn) and oxygen (O), whereas the spectrum of Z also showed the presence of sulfur (S) with a measured content of 2.93 wt%. However, no sulfur-containing crystalline phases were detected in the XRD patterns. This observation suggests that sulfur is unlikely to be incorporated into the bulk ZnO lattice and may instead be associated with non-structural surface species, residual impurities, or contamination introduced during sample preparation or analysis. Therefore, the detected sulfur is not expected to significantly influence the crystal structure of the synthesized ZnO material. The quantitative elemental compositions of SAZ, PZ, and Z are summarized in the inset tables of Fig. 7d–f.

**Fig. 8:**
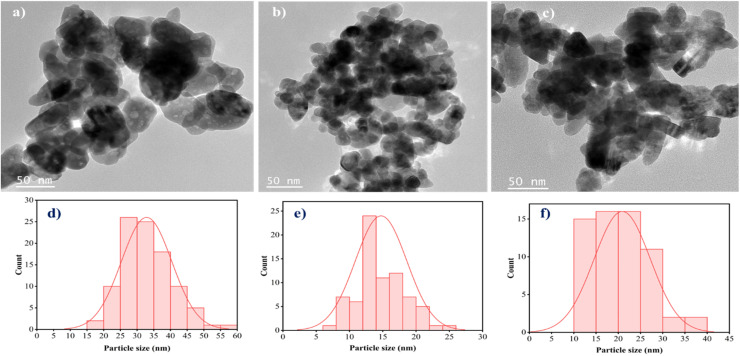
TEM images of SAZ (a), PZ (b) and Z (c) distribution of particle sizes of SAZ (d), PZ (e) and Z (f).

The morphology and particle size of the Z, SAZ, and PZ samples were examined by TEM. The SAZ sample (Fig. 8a) exhibited rod-like nanoparticles with an average particle size of 32.28 nm. In contrast, the PZ sample (Fig. 8b) consisted mainly of near-spherical nanoparticles with a markedly smaller average particle size of 14.74 nm. The Z sample (Fig. 8c) also displayed predominantly rod-like particles, with an average size of 20.87 nm, intermediate between those of SAZ and PZ. These observations were consistent with the SEM results. Moreover, the particle sizes determined from TEM were larger than the corresponding crystallite sizes estimated from XRD analysis, which may be attributed to particle aggregation and the presence of polycrystalline domains.

**Fig. 9:**
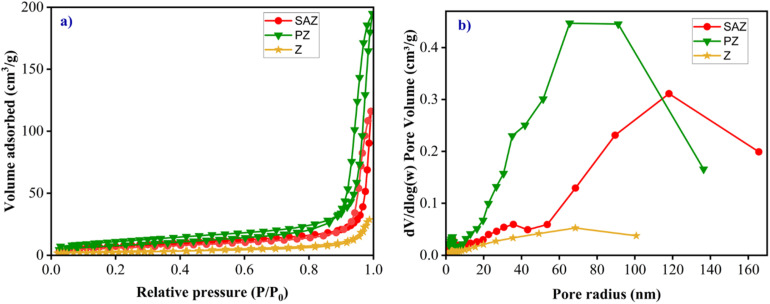
(a) The N_2_ adsorption–desorption isotherms of SAZ, PZ and Z; (b) the pore-size distribution curve of SAZ, PZ and Z.

The surface area and pore distribution of SAZ, PZ and Z were evaluated using BET isotherm analysis as shown in Fig. 9. The specific surface areas of SAZ, PZ and Z corresponded to 25, 40 and 9 m^2^ g^−1^. The SAZ sample showed a moderate surface area of 25 m^2^ g^−1^, which could be attributed to its smaller, more uniform quasi-spherical nanoparticles and more compact but homogeneous aggregation state. Notably, the PZ sample exhibited the highest BET surface area of 40 m^2^ g^−1^, corresponding well with its smallest primary particle size and the presence of abundant interparticle voids forming a hierarchical porous structure. The loose packing of near-spherical nanoparticles enhances surface accessibility and facilitates gas adsorption. In contrast, the Z sample had the lowest surface area, which was consistent with the irregular rod-like morphology observed in the SEM image of Z. The pronounced particle growth and aggregation reduced the accessible surface area by limiting the number of exposed active sites. These results reflected the evolution in particle size, morphology and aggregation behaviour, as evidenced by SEM analysis.

The BJH pore size distribution curves revealed that all samples exhibited mesoporous characteristics. The SAZ sample revealed a broad pore size distribution extending into the macroporous region, which could be attributed to the partial templating effect of organic compounds. Among the investigated samples, PZ exhibited the narrowest pore-size distribution in the range of 40–60 nm together with the highest pore volume. These observations suggest that the presence of physcion influenced nanoparticle growth and assembly during synthesis, contributing to the formation of a more homogeneous mesoporous architecture.

In particular, the hierarchical mesoporous structure of PZ provides interconnected diffusion pathways that facilitate the transport of doxycycline molecules from the bulk solution to photocatalytically active ZnO surfaces. The relatively uniform mesopore network and higher specific surface area may improve the accessibility of active interfacial sites while reducing diffusion limitations within aggregated nanoparticles. Such mesoporous channels may be especially favorable for the transport of relatively large doxycycline molecules during photocatalytic degradation. The porous framework may also promote the outward diffusion of reaction intermediates and degradation products from the catalyst surface, thereby helping to maintain efficient interfacial mass transfer throughout the reaction process. Collectively, these structural characteristics likely improve contact between doxycycline molecules and reactive surface species, which may contribute to the enhanced photocatalytic performance observed for PZ.

These results are similar to previous reports that the use of single organic compounds such as puerarin,^39^ rutin^38^ and quercetin^41^ enables better control over size, morphology and surface properties of ZnO NPs compared to the use of plant extracts in the synthesis process. These pure organic compounds facilitate controlled nucleation and growth, forming nanoparticles with greater uniformity, smaller size and higher surface area.

**Fig. 10:**
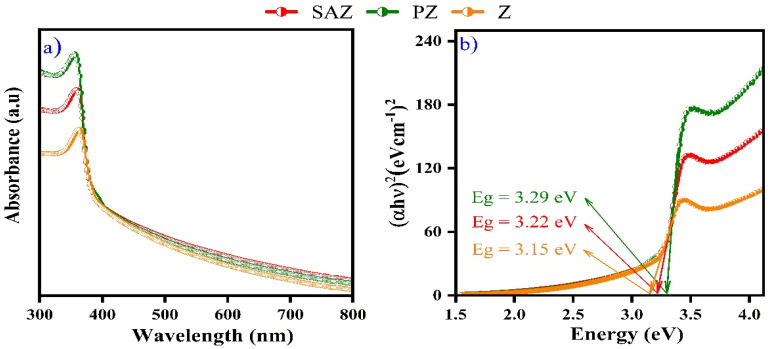
(a) UV-vis absorbance spectra of SAZ, PZ and Z, (b) band gap determination in accordance with absorption measurements of SAZ, PZ and Z.

To examine the influence of synthesis conditions on the optical properties of the prepared ZnO nanoparticles, diffuse reflectance UV-vis spectroscopy was performed. The UV-vis DRS spectra of the SAZ, PZ, and Z samples are presented in Fig. 10a. All samples exhibited a pronounced absorption edge in the near-UV region around 360–380 nm, characteristic of the intrinsic band-edge absorption of ZnO.

In addition to the absorption edge, a broad absorption tail extending throughout the visible region (400–800 nm) was observed for all samples. The intensity and spectral profile of this visible-light absorption differed among the synthesized materials, with PZ displaying the strongest absorption response and Z exhibiting the weakest. These differences suggest that the synthesis environment influences not only the crystallization behavior of ZnO nanoparticles but also their optical response.

The extended absorption observed in the visible region may be associated with modifications in the electronic structure arising from surface-related states, structural imperfections, and defect-associated electronic levels within semiconductor nanomaterials. Variations in the intensity and extent of this absorption feature suggest differences in the local electronic environments of the synthesized ZnO nanoparticles. Such changes may influence the optical absorption behavior of the materials and are consistent with the differences in optical bandgap energies discussed in the following section.

Within the limits of the optical absorption data, the observed spectral differences are consistent with subtle variations in the electronic structure of the synthesized ZnO nanoparticles. Further insight into the origin of these variations is provided by the bandgap, photoluminescence, and XPS analyses presented subsequently.

To evaluate the influence of synthesis conditions on the optical properties of the prepared materials, the optical bandgap energy (*E*_g_) was estimated using the Tauc method (Fig. 10b). The Tauc relationship for a direct bandgap semiconductor is expressed as:

4 (*αhν*)^2^ = *A*(*hν* − *E*_g_)

where *A* is a proportionality constant and *E*_g_ represents the optical bandgap energy. The bandgap values were determined by extrapolating the linear region of the (*αhν*)^2^*versus hν* plots to the energy axis. The estimated bandgap energies for SAZ, PZ, and Z were 3.22, 3.29, and 3.15 eV, respectively. Although the differences were relatively small, they were consistently observed among the analyzed samples, indicating subtle variations in the electronic and defect-related structure of the ZnO nanoparticles arising from differences in the synthesis environment.

The comparatively wider bandgap observed for PZ may be associated with combined differences in crystallinity, surface states, and nanoscale structural characteristics resulting from the synthesis route. In contrast, the slightly lower bandgap of sample Z may reflect a greater contribution from structural disorder and defect-related surface states. Such variations are commonly observed in semiconductor nanomaterials and are often influenced by the combined effects of local lattice environments, defect populations, crystallinity, and surface characteristics.

The observed bandgap trend is also consistent with the structural and morphological differences identified by XRD, SEM, TEM, and BET analyses. In particular, PZ exhibited smaller crystallite and particle sizes together with a higher specific surface area compared with Z, indicating that the synthesis route influences not only nanoparticle morphology and crystallization behavior but also the optical properties of the resulting ZnO materials.

At the same time, the relatively small magnitude of the bandgap variation suggests that no single factor is solely responsible for the observed behavior. Instead, the optical properties likely arise from the combined influence of structural disorder, surface states, local lattice environments, and nanoscale structural features. Additional insight into these factors is provided by the photoluminescence and XPS analyses discussed in the following sections.

**Fig. 11:**
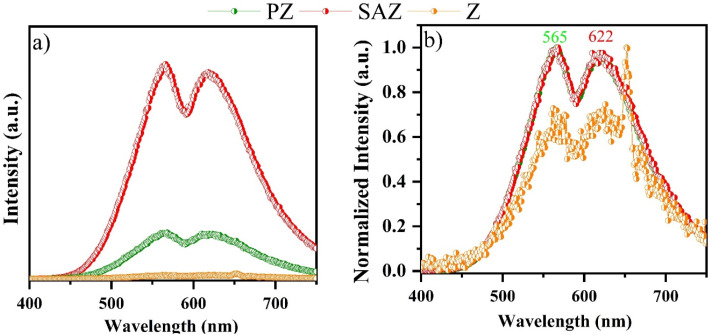
(a) Photoluminescence (PL) spectra and (b) normalized PL spectra of the SAZ, PZ, and Z samples recorded at room temperature under an excitation wavelength of 325 nm.

The photoluminescence (PL) spectra of the synthesized ZnO nanoparticles, recorded at room temperature with an excitation wavelength of 325 nm, are presented in Fig. 11a. All samples exhibited broad visible emission extending from approximately 400 to 750 nm, with two dominant emission bands centered near 565 and 622 nm. Emissions within this spectral region are commonly associated with defect-related electronic states in ZnO and are generally attributed to radiative recombination processes involving intrinsic lattice defects and surface defect environments.

Because the dominant PL emissions were located in the visible region rather than in the near-band-edge ultraviolet region, the observed luminescence is predominantly associated with defect-related electronic states and defect-mediated radiative recombination processes. Therefore, differences in PL intensity among the samples should not be interpreted solely as differences in electron–hole recombination rates. Instead, the PL behavior reflects variations in defect populations, surface states, and local electronic environments generated during synthesis. Such defect-related states may participate in charge trapping and interfacial charge-transfer processes and can therefore influence photocatalytic behavior in a more complex manner than simple band-to-band radiative recombination alone. Marked differences in PL intensity were observed among the synthesized materials. The SAZ sample exhibited the strongest emission, whereas the PL intensity of sample Z was markedly quenched. The PZ sample displayed an intermediate response. These variations suggest that the synthesis route influences both the population and distribution of radiative recombination centers within the ZnO nanoparticles. Since PL emission originates from the recombination of photogenerated charge carriers, differences in emission intensity may reflect variations in defect chemistry, surface states, and local electronic environments generated during nanoparticle formation.

For a more direct comparison of the emission profiles, the normalized PL spectra are presented in Fig. 11b. The spectral shapes of the SAZ and PZ samples were broadly similar, indicating comparable distributions of the dominant emissive states. By contrast, sample Z exhibited a noticeably different spectral profile, suggesting a distinct defect environment relative to the green-synthesized materials.

The observed differences in PL behavior are consistent with the structural and morphological characteristics identified by XRD, SEM, TEM, and BET analyses. The PZ sample possessed comparatively smaller particle dimensions together with a higher specific surface area, whereas SAZ and Z exhibited different crystallization and aggregation characteristics. Such structural variations are expected to modify the relative contribution of surface-associated electronic states and defect environments, thereby influencing the radiative recombination behavior of the materials.

The lower PL intensity observed for PZ may also be related to its distinct morphological characteristics. Compared with SAZ, PZ consisted of smaller and more uniformly distributed quasi-spherical nanoparticles, together with a higher specific surface area and a more homogeneous mesoporous architecture. Such structural features may shorten the migration distance of photogenerated electrons and holes to the particle surface, thereby reducing the probability of charge-carrier recombination within the ZnO particles. The larger accessible interfacial area may further facilitate charge separation through more efficient participation of surface redox processes. In this context, the reduced PL intensity of PZ is consistent with lower radiative recombination and improved utilization of photogenerated charge carriers. Similar morphology-dependent charge-transfer and photocatalytic behaviors have been reported for ZnO nanostructures with different shapes and aspect ratios.^42,43^ Direct verification of charge-transfer dynamics, however, would require dedicated techniques such as photocurrent measurements, electrochemical impedance spectroscopy, or time-resolved spectroscopic analyses.

From the perspective of photocatalytic performance, lower PL intensity is frequently associated with a reduced probability of radiative charge-carrier recombination under photoexcitation. Nevertheless, PL measurements primarily reflect radiative recombination processes and should not be interpreted in isolation when assessing photocatalytic activity. Photocatalytic performance is governed by multiple factors, including surface area, particle morphology, light-harvesting capability, defect chemistry, and charge-carrier dynamics. Within this framework, the superior photocatalytic activity of the PZ sample during doxycycline degradation is most reasonably attributed to the combined influence of its favorable structural characteristics, enhanced surface area, and moderated recombination behavior rather than PL intensity alone.

The broad visible emissions observed in the PL spectra are generally attributed to defect-related electronic states in ZnO. In addition to intrinsic lattice and surface defects, residual physcion-derived organic species remaining on the nanoparticle surface may modify the local electronic environment through interactions involving hydroxyl and quinone-type carbonyl functionalities. Such interactions could partially alter surface defect states and defect-mediated radiative recombination pathways, thereby influencing the observed PL intensity and overall emission characteristics. However, the currently available data do not permit direct assignment of specific emission bands, spectral features, or recombination processes to residual physcion-derived species. Accordingly, the PL behavior is interpreted primarily in terms of defect-related emissions associated with ZnO, while residual organic species are considered a plausible secondary factor that may influence the local surface defect environment and related photophysical processes.

**Fig. 12:**
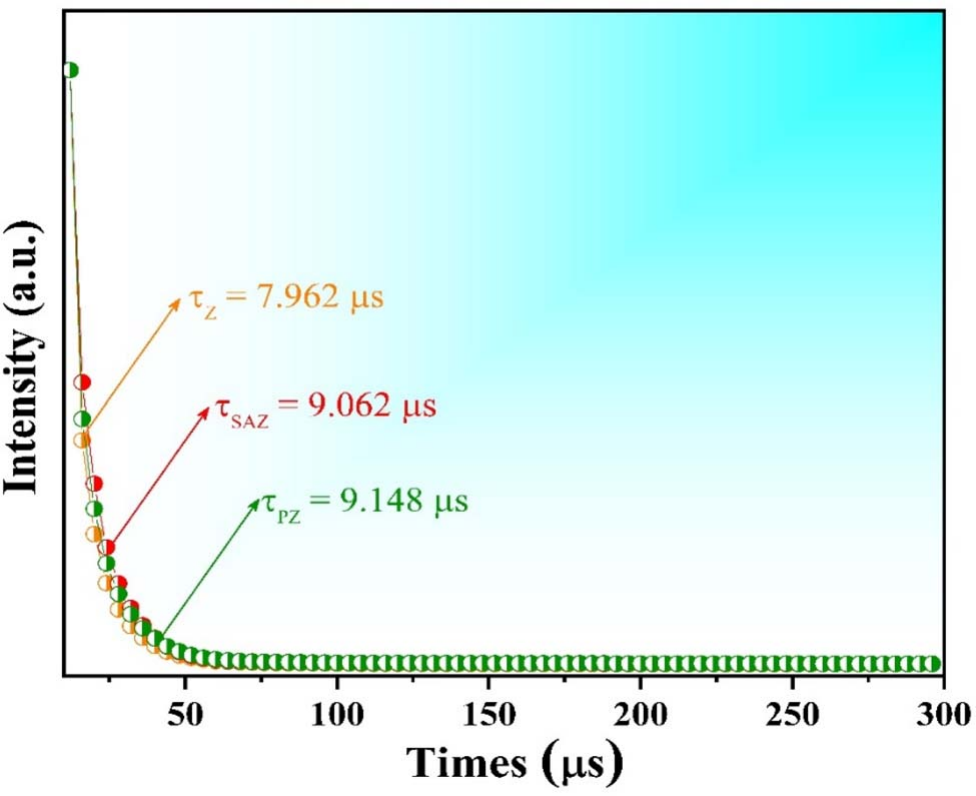
Time-resolved fluorescence spectra of Z, PZ and SAZ.

To evaluate the photocatalytic ability of samples, the lifetime of their excited electrons is crucial. Pure ZnO samples have short lifetimes, which is unfavorable for photocatalysis. Therefore, ZnO is often combined with other components to extend the lifetime of excited electrons, thereby improving the photocatalytic efficiency of the material. Based on that, we conducted time-resolved fluorescence spectroscopy measurements of the aforementioned material at an excitation wavelength of 325 nm and an emission wavelength of 565 nm. The results, as shown in Fig. 12, indicated that fluorescence decayed curve over time fits well with a double-exponential function:5
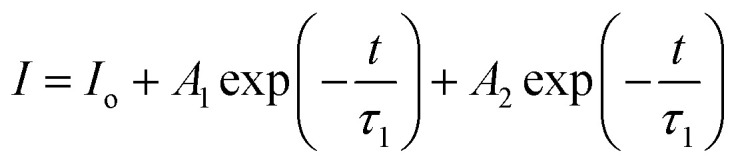
where *t* is the time, *I*(*t*) is the luminescence intensity at time *t*, *A*_1_ and *A*_2_ are constants, and *τ*_1_, *τ*_2_ are exponential components of the decay time. The value of the average lifetime τ* can be calculated using the following formula (2):6
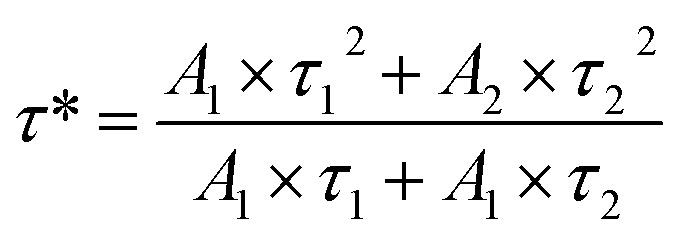


The average fluorescence lifetimes of SAZ, PZ, and Z were determined to be 9.065, 9.149, and 7.962 µs, respectively. The slightly longer lifetime observed for PZ suggests differences in charge-carrier relaxation behavior relative to the other samples. Extended excited-state lifetimes may facilitate charge separation and increase the probability of interfacial charge-transfer processes before recombination occurs. Although the differences among the samples are relatively small, the TRPL results are consistent with the enhanced photocatalytic activity observed for PZ. These observations suggest that charge-carrier dynamics may contribute, together with structural and surface-related factors, to the photocatalytic behavior of the synthesized ZnO materials.

**Fig. 13:**
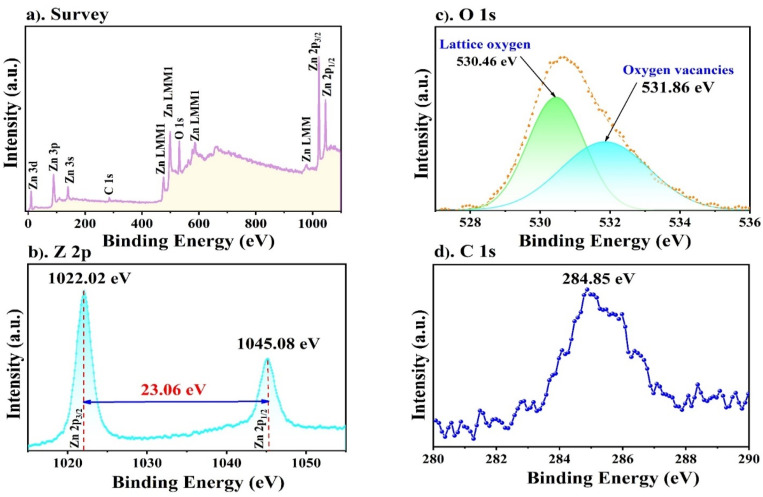
(a) XPS spectra of the ZnO NPs within the PZ sample, (b) high-resolution XPS spectra of Zn and O, (c) O 1s spectrum, (d) C 1s spectrum.

X-ray photoelectron spectroscopy (XPS) was employed to examine the surface chemical states and elemental environments of the PZ sample. The survey spectrum shown in Fig. 13a contains the characteristic Zn and O signals together with Zn LMM Auger features, consistent with the presence of ZnO-related species in the synthesized nanoparticles. All binding energies were calibrated against the adventitious C 1s signal at 284.85 eV.

A closer examination of the Zn region (Fig. 13b) reveals two well-resolved peaks at 1022.02 and 1045.08 eV. These correspond to the Zn 2p_3/2_ and Zn 2p_1/2_ components, respectively. These binding energies are typical of Zn^2+^ incorporated within the ZnO lattice. The measured spin–orbit splitting of 23.06 eV matches well with values reported for crystalline wurtzite ZnO. This observation is consistent with zinc occurring predominantly in the divalent state, with no evidence of detectable metallic Zn species.

Additional information regarding the oxygen environment was obtained from the high-resolution O 1s spectrum (Fig. 13c). Deconvolution yielded two contributions centered at 530.46 and 531.86 eV. The lower-binding-energy component is assigned to lattice oxygen (O_latt) associated with Zn–O bonds in the crystal lattice. The higher-binding-energy contribution is commonly linked to oxygen-vacancy-related species and other defect-associated surface oxygen environments. This interpretation aligns closely with the PL behavior of the PZ sample, where broad visible emission bands centered near 565 and 622 nm were observed. Such emissions are widely associated with defect-related electronic states in ZnO nanostructures. Considered together, PL and XPS observations are consistent with the presence of defect-rich surface environments that may participate in interfacial charge-transfer processes and influence charge-carrier recombination dynamics.

The C 1s signal detected at 284.85 eV (Fig. 13d) originated primarily from adventitious surface carbon and served as the reference for charge correction.

The detected C 1s signal was relatively weak, and the available XPS data did not permit reliable quantification of residual carbon species on the nanoparticle surface. No distinct spectroscopic features indicative of significant carbonaceous surface species were identified in the acquired spectra. Therefore, although the presence of trace residual carbon species cannot be completely excluded, the available evidence suggests that any such species, if present, are likely limited to minor surface amounts within the detection limits of the present analysis.

While the XPS analysis clearly establishes the Zn^2+^ oxidation state and the characteristic oxygen environments of ZnO nanoparticles, the acquired spectra do not allow a distinct Zn–O–C component to be resolved unambiguously. Consequently, the present XPS data do not provide direct experimental evidence for the existence of specific C–O–Zn linkages. In this context, the proposed involvement of physcion-derived oxygen-containing functionalities cannot be verified through XPS alone. Nevertheless, when evaluated alongside the FTIR and HPLC results, the XPS findings remain compatible with the participation of hydroxyl and carbonyl groups from physcion during ZnO nanoparticle formation. The proposed C–O–Zn interaction is therefore best viewed as a chemically plausible coordination pathway supported by converging spectroscopic observations rather than as a bonding configuration that can be established conclusively from the present dataset.

### 3.3. Photocatalytic degradation of doxycycline

The photocatalytic performance of SAZ, PZ, and Z was evaluated through the degradation of doxycycline (DOX) under UV irradiation in the presence of the photocatalyst. Prior to illumination, all samples exhibited relatively low adsorption capacities, with DOX removal after 30 min of dark equilibration reaching 6.08%, 8.11%, and 4.06% for SAZ, PZ, and Z, respectively. Upon UV irradiation, the degradation efficiency increased progressively with reaction time, following the order Z < SAZ < PZ. After 150 min, DOX removal efficiencies of 65.51%, 75.12%, and 81.20% were achieved for Z, SAZ, and PZ, respectively.

Dark-adsorption experiments accounted for only a limited portion of the overall DOX removal observed under UVA irradiation. When normalized to the total removal efficiency, adsorption contributed approximately 6.2%, 8.1%, and 10.0% for Z, SAZ, and PZ, respectively, whereas the removal occurring during the irradiation stage accounted for the remaining 93.8%, 91.9%, and 90.0%. These results indicate that adsorption alone accounted for only a minor fraction of the overall DOX removal. Most removal occurred during the UVA irradiation stage in the presence of the photocatalyst. The role of adsorption appeared largely interfacial in nature. Initial accumulation of DOX molecules near the catalyst surface may have facilitated subsequent photodegradation by increasing the probability of interaction between the adsorbed substrate and photoactive ZnO sites during irradiation.

The superior photocatalytic activity of PZ is unlikely to originate from a single physicochemical parameter but rather from the combined influence of several structural, surface, and electronic characteristics. XRD analysis revealed reduced crystallite dimensions for the bio-assisted samples relative to Z, while SEM and TEM observations demonstrated a more homogeneous particle distribution and lower aggregation tendency. Such structural characteristics may improve the accessibility of catalytically active surface regions and facilitate interfacial photocatalytic processes.

BET analysis further showed that PZ possessed the highest specific surface area and pore volume among the synthesized materials. The developed mesoporous architecture is expected to provide an enlarged catalyst–pollutant interfacial contact area and facilitate mass transport within the porous structure, thereby favoring more effective utilization of accessible catalytic sites during the degradation process.

The optical and spectroscopic analyses provide additional context for interpreting the photocatalytic behavior of the synthesized materials. UV-vis diffuse reflectance spectra revealed stronger light absorption for PZ relative to sample Z. Optical bandgap analysis further showed that PZ possessed a slightly wider bandgap (3.29 eV) than SAZ (3.22 eV) and Z (3.15 eV), reflecting subtle differences in the electronic environments of the synthesized ZnO nanoparticles. PL measurements indicated a comparatively weaker visible emission intensity for PZ than for SAZ, whereas XPS analysis identified defect-associated oxygen environments within the ZnO structure. Considered collectively, these observations are consistent with differences in the surface and electronic environments of the synthesized materials that may influence photocatalytic charge-transfer behavior.

FTIR analysis confirmed the presence of oxygen-containing surface functionalities associated with the green synthesis route, while HPLC results demonstrated substantial consumption and transformation of physcion during nanoparticle synthesis. These bio-derived surface species may modify the interaction between doxycycline molecules and the catalyst surface and contribute to the distinct photocatalytic behavior observed for SAZ and PZ relative to Z.

Taken together, the enhanced photocatalytic performance of PZ can be understood as the result of synergistic effects arising from favorable crystallite characteristics, improved particle dispersion, higher specific surface area, developed mesoporosity, modified surface chemistry, and differences in the local electronic environment of the material. Collectively, these characteristics provide a coherent explanation for the superior photocatalytic performance of PZ during doxycycline degradation.

The photodegradation of DOX by SAZ, PZ, and Z followed pseudo-first-order kinetics, as evidenced by the linear relationship between ln(*C*_*t*_/*C*_0_) and irradiation time together with the high correlation coefficients obtained from the kinetic fitting. The apparent rate constants were calculated to be 0.0093, 0.0108, and 0.0070 min^−1^ for SAZ, PZ, and Z, respectively, confirming the superior photocatalytic performance of PZ relative to the other materials.^40^

**Fig. 14:**
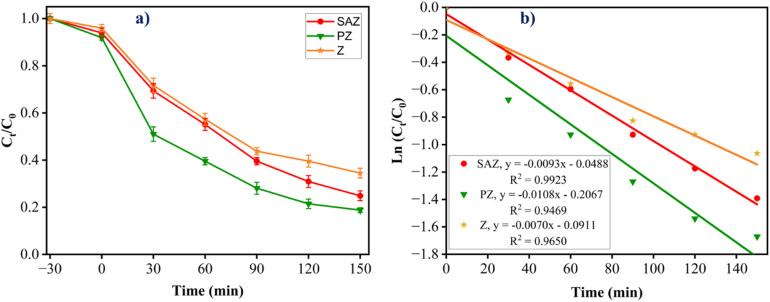
Photodegradation of DOX by SAZ, PZ and Z: (a) *C*_*t*_/*C*_0_*versus* reaction time; (b) ln(*C*_*t*_/*C*_0_) *versus* reaction time.

To contextualize the photocatalytic performance of PZ, its activity toward doxycycline degradation was compared with previously reported ZnO-based photocatalysts, as summarized in Table 1. PZ exhibited a pseudo-first-order rate constant of 0.0108 min^−1^ together with 81% degradation efficiency, indicating competitive photocatalytic performance relative to several reported ZnO-based systems. Direct comparison among different studies should be interpreted with caution because photocatalytic performance is strongly influenced by factors such as pollutant concentration, catalyst dosage, irradiation source, and reaction conditions. In particular, variations in the initial doxycycline concentration may substantially affect degradation efficiency and apparent kinetic behavior (Fig. 14).

**Table 1:** Comparation of DOX photodegradation of ZnO NPs

Catalyst	Degradation efficiency (%)	Concentration of DOX (mg L^−1^)	*k* (min^−1^)	Ref.
Polymer-ZnO composite	75	50	0.0040	44
ZnO NPs	87	40	0.0036	21
Lavender-derived ZnO/biochar	63	25	0.0032	45
ZnO NPs	68	50	0.0062	46
PZ	81	10	0.0108	This study

**Fig. 15:**
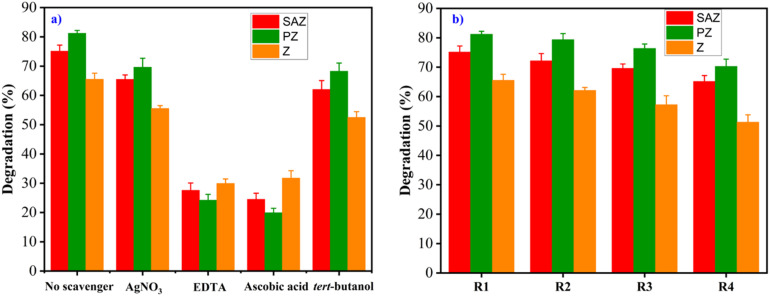
(a) Effect of different scavengers on DOX photodegradation by SAZ, PZ and Z; (b) recycling of SAZ, PZ and Z.

To elucidate the reactive species involved in DOX degradation, radical scavenging experiments were conducted using AgNO_3_, EDTA, ascorbic acid, and *tert*-butanol as scavengers for e^−^, h^+^, O_2_˙^−^, and ˙OH, respectively (Fig. 15a). The addition of EDTA and ascorbic acid resulted in a pronounced decrease in photocatalytic efficiency for all samples, whereas AgNO_3_ and *tert*-butanol produced comparatively smaller effects. These observations suggest that photogenerated holes (h^+^) and superoxide radicals (O_2_˙^−^) play dominant roles in the photocatalytic degradation of DOX under the investigated conditions, while electrons and hydroxyl radicals make comparatively smaller contributions to the overall process.^47^ Catalyst reusability was examined through consecutive recycling experiments as an initial measure of operational stability. As presented in Fig. 15b, the photocatalyst was recovered and reused in four successive cycles. DOX removal remained at a relatively high level throughout the recycling tests, although a gradual loss of activity became apparent with repeated use. The decline was more pronounced after the fourth cycle, which may reflect the progressive depletion of accessible active sites together with incomplete regeneration of the catalyst surface between cycles.

While these observations support acceptable short-term reusability, they do not provide a sufficient basis for judging long-term durability. A more rigorous assessment will require a substantially larger number of recycling cycles, accompanied by evaluation of photocorrosion, structural evolution, and possible Zn leaching under extended operating conditions.

**Fig. 16:**
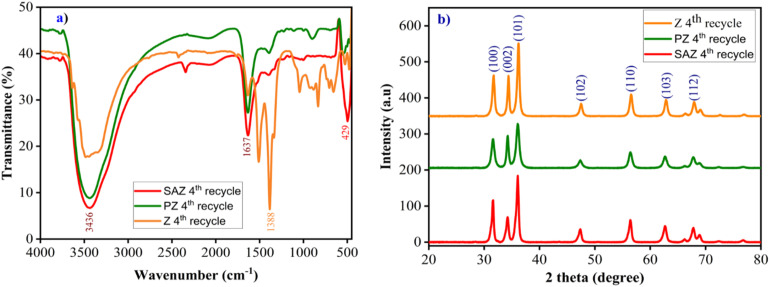
(a) FTIR spectra of SAZ, PZ and Z after 4th recycling; (b) XRD of SAZ, PZ and Z after 4th recycling.

To further examine catalyst stability, FTIR spectra and XRD patterns of the recovered samples were recorded after the fourth cycle (Fig. 16a and b). The FTIR spectra and XRD patterns remained largely unchanged, with only minor variations in peak intensity. The absence of major spectral or structural changes suggests that the principal crystal framework and surface functionalities of the catalysts were largely preserved throughout the recycling experiments, indicating acceptable structural stability under the investigated recycling conditions.

**Fig. 17:**
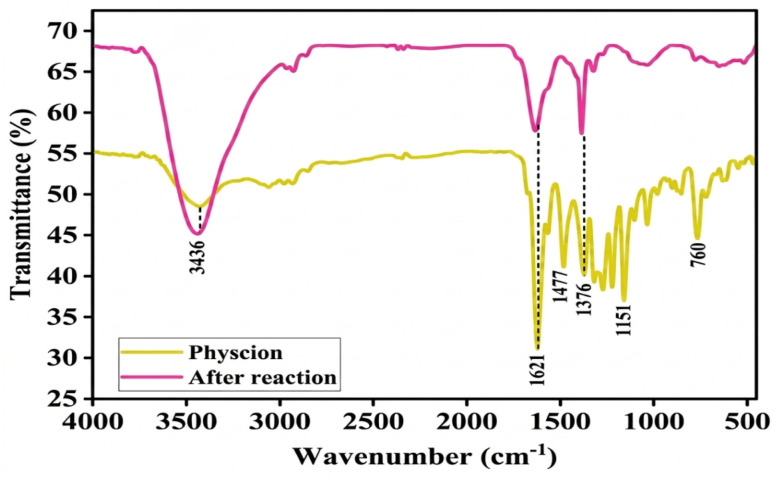
FTIR spectra of DOX and mixture after photodegradation of DOX.

FTIR spectroscopy was used as a preliminary tool to assess functional-group changes occurring during photocatalytic degradation of doxycycline. The FTIR spectrum of DOX and products after photodegradation of DOX using PZ was shown in Fig. 17. The FTIR spectra of DOX exhibited an absorption peak at 3338 cm^−1^, associated for stretching vibrations of –OH and N–H group. The absorption peaks at 1633 cm^−1^ and 1579 cm^−1^ were assigned to the stretching vibration of C=O in amide I and the bending vibration of the N–H group, respectively.

In addition, absorption peaks at 1331 and 1044 cm^−1^ were attributed to the vibration of C–N and C–O bonds.^48^ However, absorption peaks at 1459 cm^−1^, associated with stretching vibration of aromatic C=C bonds, had disappeared, suggesting interaction or modification of aromatic groups. Similar results were observed when photocatalytic degradation of DOX using different materials, such as ZnO/NiCo_2_O_4_ QDs-OVs using *Punica granatum* fruit peel extract^21^ and (La_2_O_3_/BiO)_2_CO_3_/Ag_3_PO_4_.^49^

After photocatalytic treatment, noticeable changes in the FTIR profile were observed. In particular, the disappearance of the band at 1459 cm^−1^, assigned to aromatic C=C vibrations, together with variations in other characteristic absorption bands, suggests substantial alteration of the molecular structure of DOX during photocatalytic degradation. Although FTIR spectroscopy does not permit unambiguous identification of individual degradation intermediates, the observed spectral changes provide evidence for extensive transformation of the parent antibiotic molecule. Similar observations have been reported for ZnO-based photocatalytic systems employed for DOX degradation.^49^

**Fig. 18:**
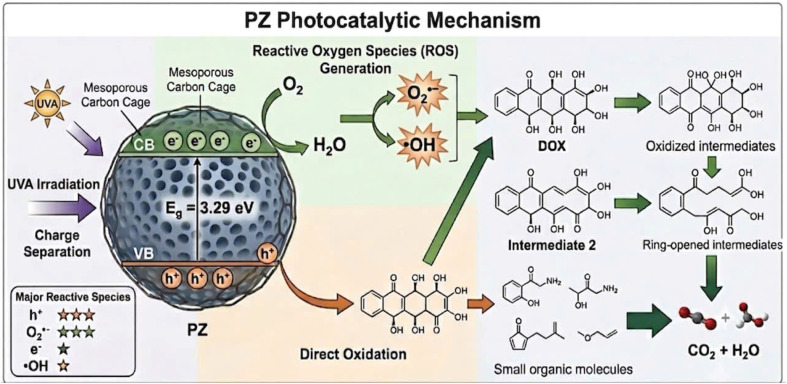
Proposed mechanism of photocatalytic degradation of DOX using PZ.

A schematic representation of the proposed photocatalytic mechanism is shown in Fig. 18. Upon UV irradiation, ZnO nanoparticles generate photogenerated electrons in the conduction band and holes in the valence band. The scavenger experiments suggest that h^+^ and O_2_˙^−^ are the principal reactive species involved in the degradation process. Electrons transferred to dissolved oxygen may promote the formation of superoxide radicals, while photogenerated holes may directly participate in oxidation reactions at the catalyst surface.

The XPS and PL analyses suggest the presence of oxygen-vacancy-related species and defect-rich surface environments in the synthesized ZnO nanoparticles. Such defect sites may facilitate the adsorption and activation of dissolved oxygen molecules and may also influence charge-carrier dynamics by acting as electron-trapping centers. These processes could favor the formation of reactive oxygen species under irradiation, particularly superoxide radicals and related secondary oxidizing species. In this context, the enhanced photocatalytic activity of PZ may arise not only from its favorable structural characteristics but also from defect-mediated interfacial processes associated with ROS generation. Direct verification of the specific role of oxygen vacancies in ROS formation, however, would require dedicated spectroscopic techniques such as EPR analysis.

The surface chemistry introduced through the green synthesis route, together with the structural and electronic characteristics discussed above, may influence interfacial charge-transfer processes and pollutant–catalyst interactions. Through a sequence of oxidation reactions, DOX molecules are progressively transformed into smaller organic species. Based on previously reported LC-MS studies of doxycycline photocatalytic degradation, demethylation, hydroxylation, deamidation, and ring-opening reactions have frequently been proposed as major transformation pathways.^49,50^ In the present study, FTIR analysis revealed substantial changes in the characteristic functional groups of doxycycline after photocatalytic treatment, indicating extensive structural transformation of the parent compound. Such observations are broadly consistent with progressive molecular transformation during photocatalysis, although the specific intermediates generated in the present system could not be identified. However, FTIR spectroscopy does not provide molecular-level identification of degradation intermediates. Therefore, definitive elucidation of the degradation pathway would require LC-MS/MS or GC-MS analysis.

### 3.4. Environmental implications

Doxycycline residues are frequently detected in hospital effluents, livestock wastewater, and pharmaceutical discharges because conventional biological treatment processes often fail to achieve complete removal of persistent antibiotics. Continuous release of these compounds into aquatic environments contributes to selective pressure for antibiotic resistance and complicates long-term water-quality management.

Within this context, the physcion-derived ZnO system developed here offers several features relevant to photocatalytic water treatment. Use of a structurally defined phytochemical during synthesis may provide greater control over nucleation and particle growth than is typically achievable with compositionally complex plant extracts. The resulting PZ material exhibited smaller crystallite dimensions, a more homogeneous mesoporous framework, and greater accessible surface area. Such structural characteristics may promote interfacial redox interactions and facilitate more efficient utilization of photogenerated charge carriers during photocatalytic treatment. Stable activity across repeated reuse cycles further supports the robustness of the physcion-mediated ZnO architecture under the investigated conditions.

Several issues nevertheless remain unresolved before practical implementation can be considered fully realistic. Photocatalytic behavior should be validated in real wastewater matrices containing dissolved organic matter, inorganic ions, and competing contaminants. Identification of transformation intermediates and evaluation of residual toxicity will also be essential to establish whether photocatalytic treatment results in a net reduction of environmental risk.

### 3.5. Limitation and future perspectives

Several limitations should be acknowledged before practical application of the proposed photocatalytic system can be considered. The present study employed UVA irradiation under controlled laboratory conditions; therefore, improving visible-light utilization of physcion-derived ZnO remains an important objective for future development. In addition, photolysis controls in the absence of catalyst were not performed, preventing quantitative separation of direct UVA photolysis from adsorption and photocatalytic degradation contributions.

Further mechanistic clarification of physcion-mediated ZnO formation is also required. Although FTIR and HPLC analyses support the participation of physcion during nanoparticle formation, the structures of the transformation-derived species could not be conclusively identified. Advanced analyses such as LC-MS, post-reaction NMR, and high-resolution XPS would provide deeper insight into the coordination and transformation processes governing ZnO formation.

Another limitation concerns the absence of direct identification of doxycycline degradation intermediates. Consequently, the degradation pathway, mineralization behavior, and toxicity evolution of transformation products remain unresolved. Future studies employing LC-MS/MS, GC-MS, TOC analysis, and residual antibacterial activity assessment will be necessary to establish the environmental safety of the photocatalytic process.

Although scavenger experiments provided preliminary evidence regarding dominant reactive species, direct investigation of reactive oxygen species generation and charge-carrier dynamics remains necessary. Techniques such as EPR spectroscopy and time-resolved spectroscopic analyses could provide more definitive mechanistic insight.

Finally, long-term operational stability and practical applicability require further evaluation. Future work should examine photocorrosion resistance, Zn leaching behavior, continuous-flow performance, and photocatalytic activity in real wastewater matrices containing dissolved organic matter, inorganic ions, and coexisting contaminants.

## 4. Conclusion

In summary, physcion, an anthraquinone isolated from *Senna alata*, emerged as a key participant in the green synthesis of ZnO nanoparticles. Evidence obtained from FTIR, HPLC, and complementary structural analyses points to its involvement in Zn^2+^ coordination, coordination-associated chemical transformation, and stabilization of the developing nanoparticle surface. Taken together, these observations support a growth process in which physcion influences nucleation, crystallite evolution, and particle assembly throughout ZnO formation.

Compared with crude extract-mediated and chemically synthesized ZnO, the physcion-derived material (PZ) displayed smaller crystallite dimensions, reduced particle size, higher specific surface area, a more homogeneous mesoporous architecture, and photoluminescence behavior indicative of lower charge-carrier recombination. Among the investigated materials, PZ also delivered the highest photocatalytic degradation efficiency toward doxycycline under UVA irradiation.

The significance of these findings extends beyond the development of an alternative green synthesis route. The results illustrate how a structurally defined anthraquinone can influence multiple stages of nanoparticle formation, from precursor coordination to crystal growth and surface evolution. In this regard, the comparison between physcion and the crude plant extract underscores the value of employing selected phytochemical mediators rather than chemically heterogeneous botanical mixtures when greater control over nanomaterial structure and photocatalytic function is desired. Such insights may prove useful in the future design of green-synthesized photocatalysts for environmental remediation applications.

## Author contributions

Khieu Thi Tam: conceptualization, investigation, writing and editing, Dang Van Thanh: conceptualization, validation and editing, Nguyen Khac Tung: formal analysis, methodology, Le Tien Ha: data curation, formal analysis, Vuong Truong Xuan: editing, writing – review, investigation, Tran Trung Hieu: conceptualization, resource, Minh: formal analysis, data curation, Cao Thanh Hai: conceptualization, resource, writing – original draft.

## Conflicts of interest

There are no conflicts of interest.

## Supplementary Material

RA-016-D6RA01923D-s001

## Data Availability

All data are provided in manuscript and supplementary information (SI). Supplementary information: spectroscopic characterization data, including the ^1^H-NMR spectrum of physcion isolated from *Senna alata* (Fig. S1) and the FTIR spectrum of the *Senna alata* extract used in the green synthesis of ZnO nanoparticles (Fig. S2). See DOI: https://doi.org/10.1039/d6ra01923d.
