# Supplementary material for: Physcion isolated from Senna alata-mediated green synthesis of ZnO nanoparticles: mechanistic and comparative evaluation for photocatalytic doxycycline degradation
Source: RSC Adv. 2026 Jul 2;16(34):32585–605. doi: 10.1039/d6ra01923d (PMC13325631; doi:10.1039/d6ra01923d)
Supplement: RA-016-D6RA01923D-s001 [file RA-016-D6RA01923D-s001.pdf]

Supplementary Information (SI) for RSC Advances.
This journal is © The Royal Society of Chemistry 2026

Supporting Information

Physcion isolated from *Senna alata*-mediated zinc oxide nanoparticles: mechanistic and comparative insights for photocatalytic doxycycline degradation

Khieu Thi Tam[1], Dang Van Thanh[2,3], Nguyen Khac Tung[2], Le Tien Ha[1], Vuong Truong Xuan[1*], Tran Trung Hieu[4], Dinh Thi Hong Minh[5], Vu Thi Hue[6], Cao Thanh Hai[1*]

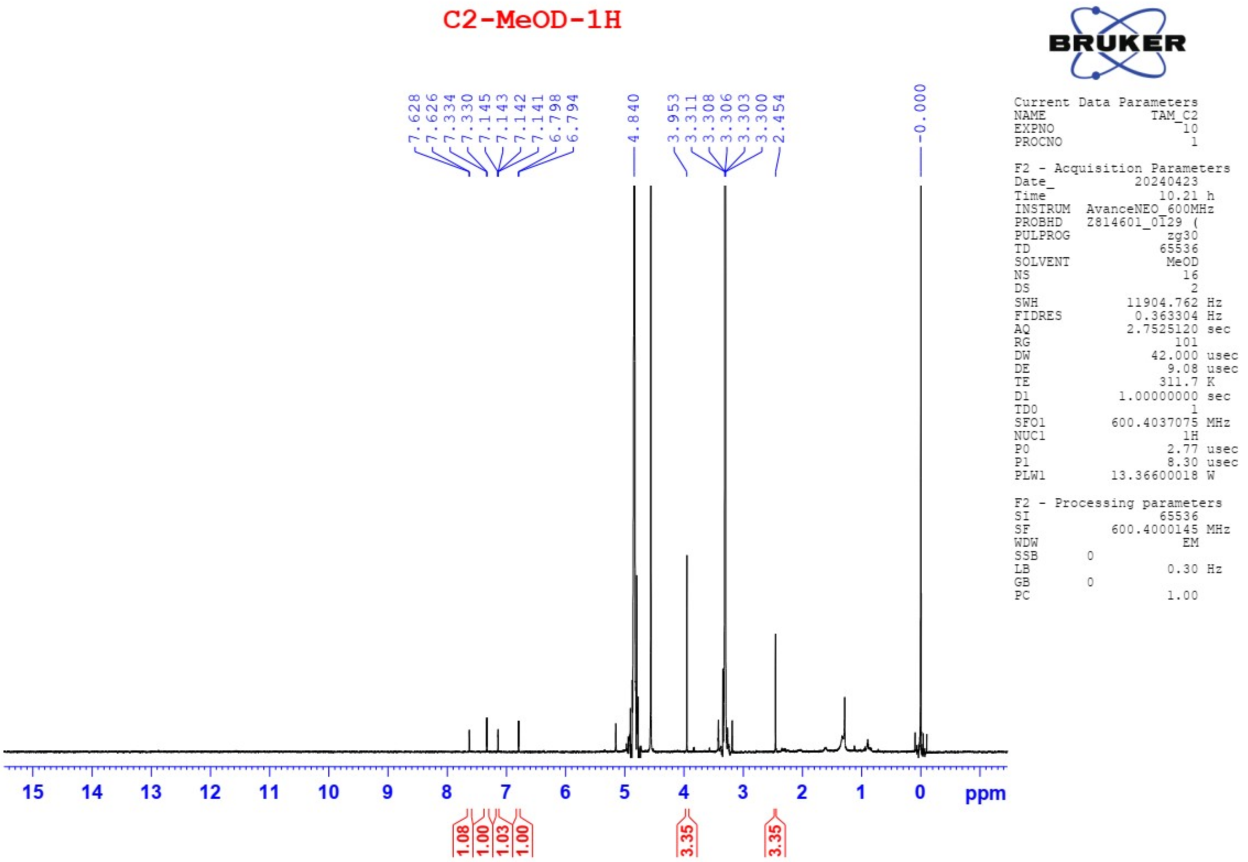

C2-MeOD-1H

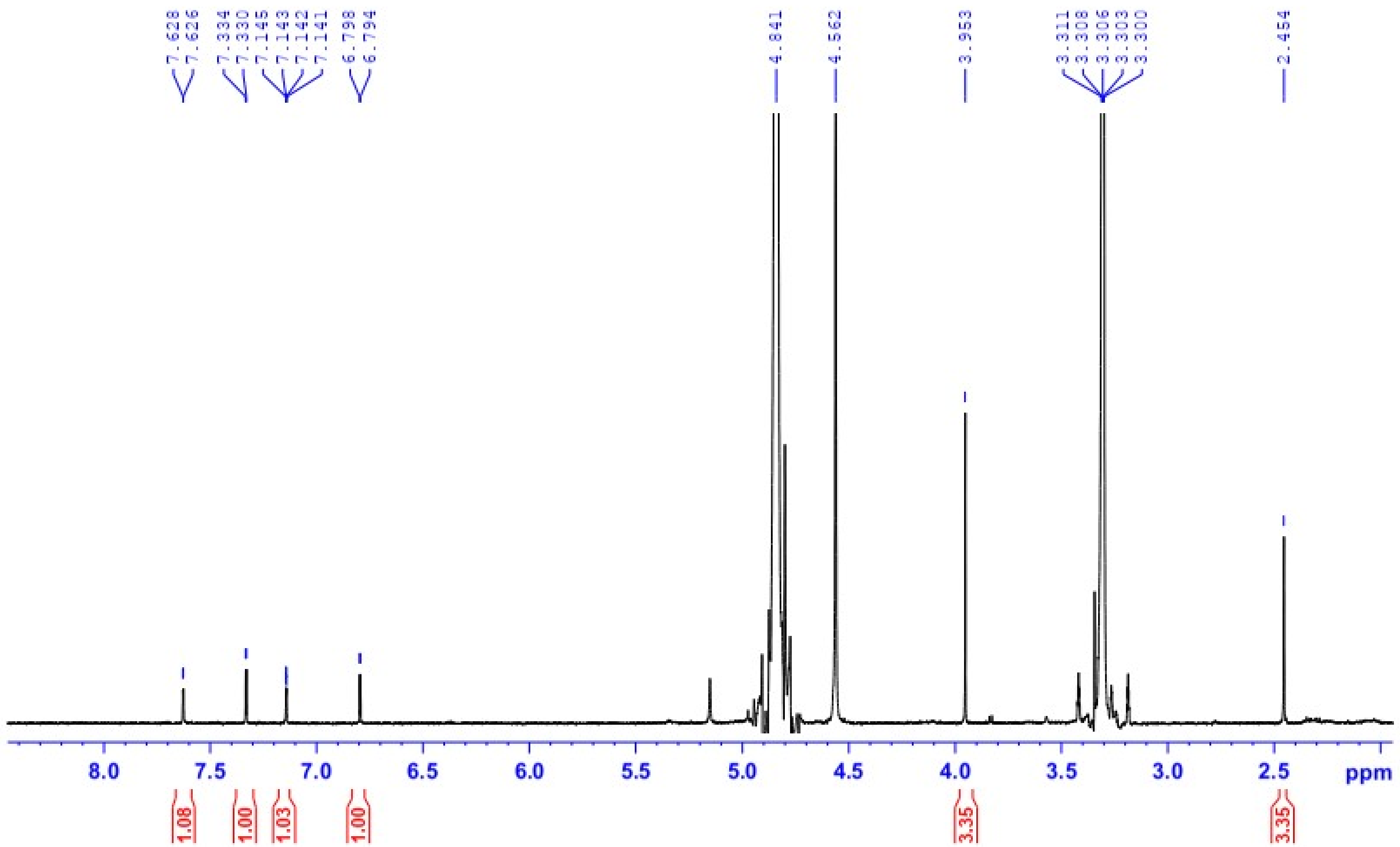
7.628
7.626
7.334
7.330
7.145
7.143
7.142
7.141
6.798
6.794
4.841
4.562
3.953
3.311
3.308
3.306
3.303
3.300
2.454
8.0
7.5
7.0
6.5
6.0
5.5
5.0
4.5
4.0
3.5
3.0
2.5
ppm
1.08
1.00
1.03
1.00
3.35
3.35

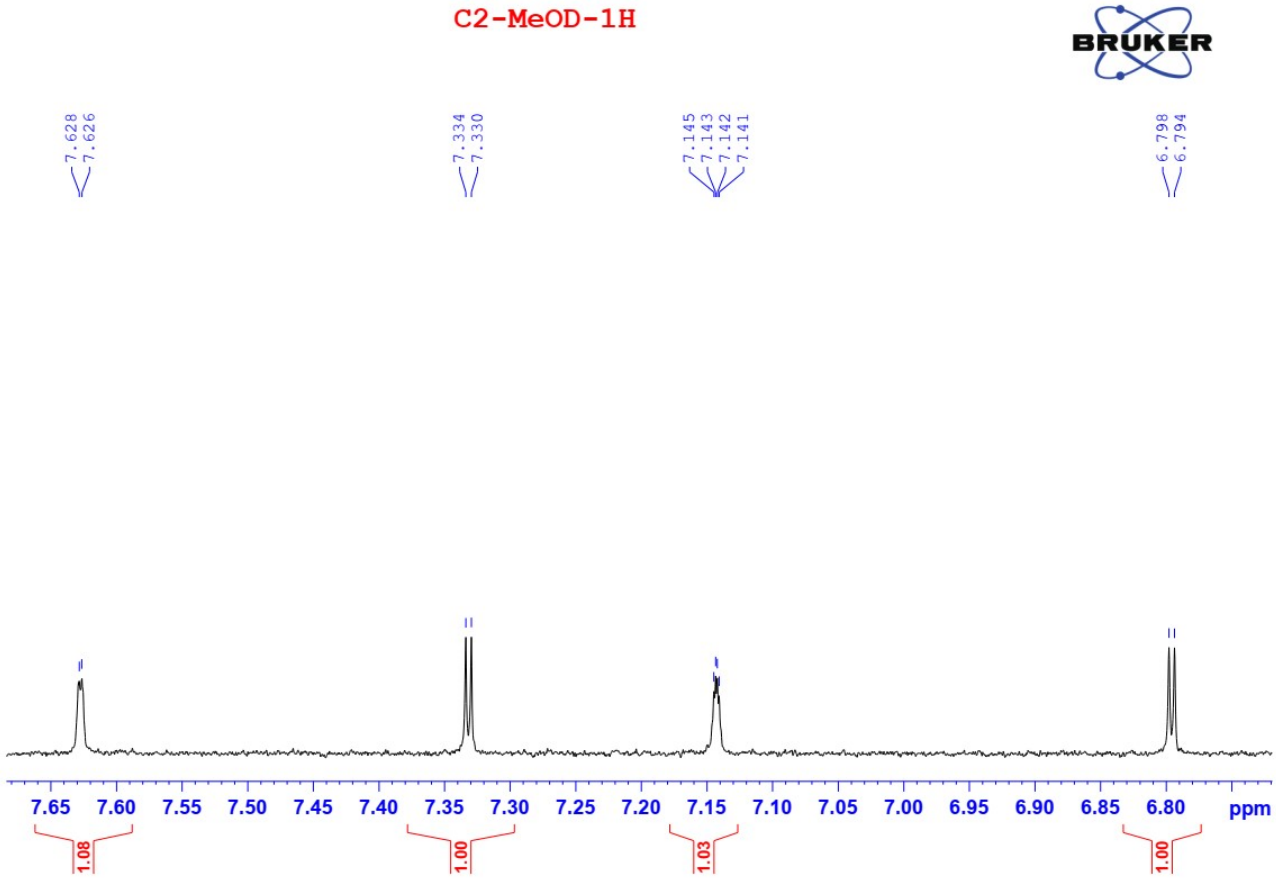

Fig. S1. 1H-NMR spectra of physcion

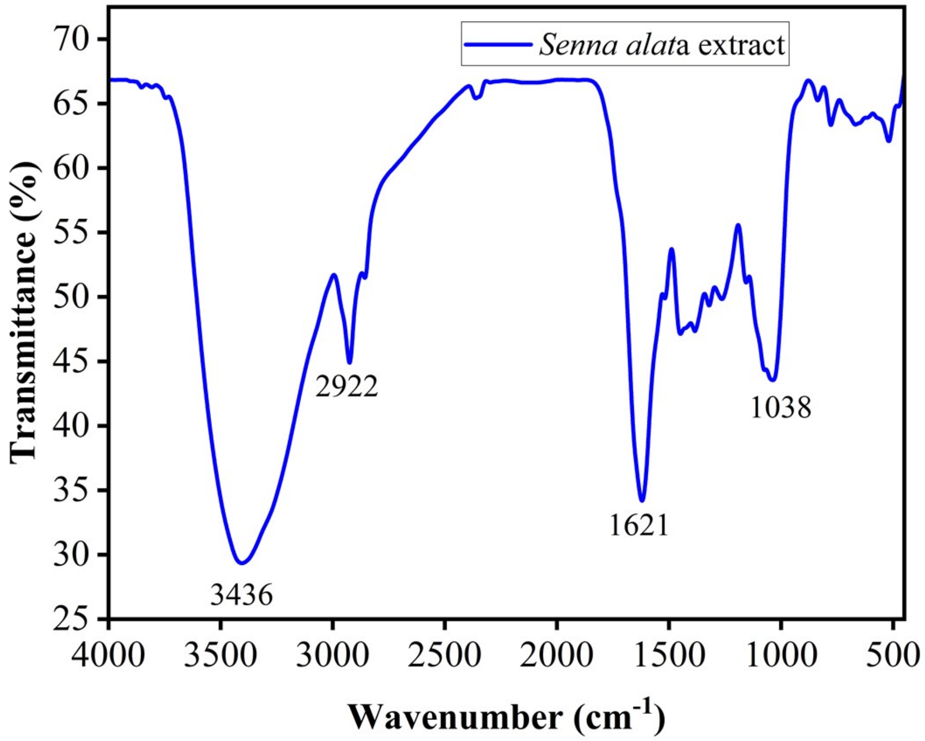

Fig. S2. FTIR spectra of *Senna alata* extract
